# NMDA receptor mediates proliferation and CREB phosphorylation in postnatal Müller glia-derived retinal progenitors

**Published:** 2009-04-10

**Authors:** Mónica Ramírez, Mónica Lamas

**Affiliations:** Departamento de Farmacobiología, Cinvestav Sede Sur, México DF, México

## Abstract

**Purpose:**

Postnatal retinal Müller glia are considered to be retinal progenitors as they retain the ability to dedifferentiate, proliferate, and differentiate to new retinal glia and neurons after injury. The proliferation and differentiation processes are coordinated by several extrinsic factors and neurotransmitters, including glutamate. Thus, the appropriate numbers and proportions of the different cell types are generated to form a functional retina during development and during injury repair. Here we analyze the changes in the proliferation of postnatal Müller glia-derived progenitors after activation of the N-methyl-D-aspartate (NMDA) glutamate receptors.

**Methods:**

Müller glia-derived progenitor cell cultures were characterized by immunocytochemistry with antibodies against the NR1 subunit of the NMDA receptor and the progenitor cell marker nestin. The effect of glutamate receptor agonists and antagonists on cell proliferation was analyzed by BrdU incorporation or Ki67 immunostaining, cell counting, and by immunolabeling of phosphorylated cAMP response element binding protein (P-CREB) transcription factor. The effect of NMDA receptor activation was analyzed in vivo by P-CREB immunohistochemistry in retinal sections of Long-Evans NMDA injected rats.

**Results:**

We show that NMDA receptor activation significantly increases the proliferation rate of Müller-glia derived progenitor cells and that this increase can be blocked by NMDA receptor antagonists. Furthermore, we show that CREB phosphorylation is induced in NMDA-treated Müller-glia derived progenitor cells in culture and that specific pharmacological inhibition of CREB phosphorylation results in a decreased number of proliferating cells. We confirmed the relevance of these observations by the analysis of retinal sections after NMDA injection in vivo where immunoreactivity to phosphorylated CREB is also increased after treatment.

**Conclusions:**

In the present study we show that NMDA receptor activation induces postnatal Müller glia-derived retinal cell progenitor proliferation and transcription factor CREB phosphorylation both in culture and in vivo. The identification of the molecular determinants of mature retinal progenitors such as transcription factor CREB and NMDA receptor-induced players should facilitate the control of growth and manipulation of progenitor cell cultures and the possible identification of the molecular mechanisms involved in progenitor self-renewal.

## Introduction

The vertebrate retina presents seven major cell types including rod and cone photoreceptors, retinal ganglion cells, horizontal cells, amacrine cells, bipolar cells, and the Müller glia. During development, multipotent retinal progenitors generate all retinal cell types [1]. Around 12 days postnatal, the mouse retina is fully developed [2]. At early stages of retinal development neurotransmitters modulate the proliferation and differentiation of progenitor cells [3]. Among them, the excitatory neurotransmitter glutamate acts as an antiproliferative factor in the developing mouse retina [4].

Unlike different regions of the adult brain and the embryonic retina [5,6] active neurogenesis has not been detected in the normal adult mammalian neural retina. However, several recent studies have demonstrated that Müller cells can acquire neurogenic potential in response to injury to the retina thus acting as latent neural stem cells in this tissue. This notion is supported by the following evidence: Muller glia undergo a proliferative response after N-methyl-D-aspartate (NMDA)-mediated neurotoxic injury to the chicken and mouse retina, and some of the progeny differentiate into neurons [7,8]. The capacity of chicken Müller glial cells to undergo a proliferative response after intraocular injection of growth factors could be also evoked [9]. Müller cells, enriched from the normal rat retina, generate clonal neurospheres capable of differentiating into functional neurons and to generate site-specific neurons upon transplantation [10]. In the zebrafish, Müller glia-derived progenitors are late retinal progenitors that generate the rod photoreceptor lineage in the postembryonic retina [11]. It has been recently shown that Müller glia in adult mice can be induced to dedifferentiate, migrate, and generate new retinal neurons and photoreceptor cells by glutamate [12].

We have previously demonstrated that differentiated Muller glia from the postnatal rat retina have functional NMDA subtypes of glutamate receptors that, upon activation, induce transcription factors and modulate gene expression [13,14]. Among them, we described that cAMP response element binding protein (CREB), a pleiotropic transcription factor that has been involved both in cell proliferation and survival [15], is phosphorylated and therefore activated, upon glutamate stimulation in these differentiated cells [13].

In light of previous discrepancies regarding the effect of NMDA receptor modulation of the proliferation of adult-derived progenitor cells from the brain [16-18] and the developing retina [4], we wanted to examine the effects of NMDA receptor agonists and antagonists on the proliferation of postnatal Müller glia-derived retinal progenitors in culture. In the present study, we show that NMDA receptor activation induces postnatal Müller glia-derived retinal cell progenitor proliferation and transcription factor CREB phosphorylation both in culture and in vivo.

## Methods

All experiments were conducted on laboratory animals treated and handled in accordance with the Association for Research in vision and Ophthalmology (ARVO) Statement for the Use of Animals for Ophthalmic and Vision Research. Adult Long-Evans pigmented rats were used in all experiments. Rats were obtained from Harlan Sprague-Dawley (Madison, Wisconsin), maintained on a 12–12 light-dark cycle and had access to standard rat chow and water ad lib and were sacrificed by decapitation.

### Cell culture

Enucleated eyes from 8- to 10-day-old Long-Evans rats were placed in Dulbecco’s Minimal Essential Medium (DMEM; Gibco BRL, Gaithersburg, MD) containing 10% fetal bovine serum (FBS) and 1:1,000 penicillin-streptomycin and stored overnight in the dark at room temperature. The eyes were incubated the next day at for 30 min at 37 °C in DMEM containing 0.1% trypsin and 70 IU/ml collagenase (Sigma Chemical Co., St. Louis, MO) following which they were transferred to 10% FBS-DMEM. Subsequently, the retina was dissected away from the rest of the tissue, and its cells were dissociated with a Pasteur pipette. Cells from two retinas were seeded onto a six well Petri dish and cultured in OptiMEM (Gibco BRL, Gaithersburg, MD) containing 4% FBS. The cultures were maintained for two weeks or until they were confluent, and then they were seeded again into 75 cm^2^ flasks until confluent.

The technique employed here for primary culture is routinely used as a source of purified Müller cells has been previously reported [19]. The absence of neuronal, fibroblastic or astrocytic contaminations in the culture has been confirmed. A detailed analysis of the purity of the cell culture obtained by this protocol showed that these cells expressed a battery of transcripts characteristic of Müller cells such as glutamine synthase (GS), cellular retinaldehide binding protein (CRALBP), vimentin, clusterin, and carbonic anhydrase, while transcripts corresponding to rod photoreceptors, bipolar cells, retinal ganglion cells, amacrine cells endothelial cells, or retinal pigmented epithelium cells were not expressed [10]. The presence of nonneurosphere-forming contaminating microglia in the monolayer culture in the range of 2%–4% has been reported [10].

In our hands, more than 96% of the cells were immunopositive for CRALBP [13]. These cultures were also immunonegative for the progenitor marker nestin, as has been previously reported [20].

Once a confluent Müller cell monolayer was obtained (around 14 days in culture), the cells were scraped and cultured in OptiMEM (Gibco BRL, Gaithersburg, MD) containing 30 ng FGF-2 (Peprotech, NJ), 30 ng EGF (Peprotech) and 100X N2 supplement (Invitrogen, Carlsbad, CA). Cells were maintained as controls or exposed to the following compounds for five days: 50, 100, and 500 μM NMDA (Sigma Chemical Co.); 50, 100, and 500 μM glutamate (Sigma Chemical Co.); 50 μM MK801 (RBI, Natick, MA); 100 µM NBQX (Tocris, Ellisville, MI), or 25 µM CREB inhibitor 2-naphthol-AS-E-phosphate (Sigma Chemical Co.).

### Immunocytochemistry

The cells were cultured on Poly-D-Lysine (Sigma Chemical Co.) coated slides for five days and fixed with 4% paraformaldehyde for 15 min or the retinal sections were washed three times in PBS (137 mM NaCl, 2.7 mM KCl, 4.3 mM Na_2_PO_4_; 1.47 mM KH_2_PO_4_) and blocked in 1% gelatin and 10% goat normal serum in PBS containing 0.3% Tween-20. The cells were stained with primary antibodies for P-CREB (1:50; Cell

Signaling, Danvers, MA), CRALBP (1:100; Affinity BioReagents, Rockford, IL), nestin (1:100; Chemicon, Temecula, CA), the NMDA receptor subunit NR1 (1:100; Pharmigen, San Diego, CA), and Ki67 (1:1,000; Millipore, Billerica, MA), which were diluted in the blocking solution before they were added. The slides were allowed to sit overnight at 4 °C. After extensive washing, the double staining was developed using a 1:1,000 dilution of Alexa 568 anti-mouse antibody and Alexa 488 anti-rabbit (Molecular Probes, Invitrogen, Carlsbad, CA). Finally, the cell nuclei were counterstained with 0.1 mg/ml 4´, 6-diamidino-2-phenylindole (DAPI) for 10 min. The slides were mounted with Vectashield (Vector Laboratories, Burlingame, CA) and observed using a fluorescence inverted microscope (Nikon E 600, Nikon, Melville, NY).

### Bromodeoxyuridine incorporation assay in culture

The Roche BrdU Labeling and detection kit (cat. number. 11296736001, Roche, Indianapolis, IN) was used according to the manufacturer's directions. Briefly, cells were cultured in chamber slides for five days and exposed to 50–100–500 μM NMDA or glutamate, 50 μ;M antagonist MK801 or 100 μM NMDA plus antagonist or medium alone in control cultures. The last day BrdU (10 mM) was added to the chamber. BrdU incorporation into newly synthesized DNA of proliferating cells was estimated by immunocytochemistry using specific monoclonal anti-BrdU and FITC (fluorescein isothiocyanate)–conjugated secondary antibodies. Approximately 300 DAPI positive cells were counted in fields from different treatments. From these, the percentage of cells that were BrdU-positive was determined. Experiments were performed in triplicate.

### Statistical analysis

Comparisons were made by ANOVA followed by the “*t*” student or Holm-Sidak procedures for posthoc comparisons. Values of p<0.05 and p<0.01 were considered significant.

### Intravitreal injection and bromodeoxyuridine incorporation assay in vivo

While under anesthesia, 21-day-old Long Evan rats were given an intravitreal injection of 2 M NMDA and 10 mM BrdU with a 30 gauge needle on a microsyringe (Hamilton, Reno, NV). The drugs were dissolved in an equal volume of 3 µl. Sham injections of 3 µl BrdU in normal saline were performed in each rat’s left eye. The rats were allowed to recover spontaneously from the anesthesia. Then they were sent back to the animal room, where they were provided with food and water ad libitum. At different times after injection (1 h, 2 h, and 1 day) the rats were euthanized using an overdose (80 mg/kg) of pentobarbithal (Anestesal, Pfizer, Mexico) injected intraperitoneally. For preparation of cryostat sections, the eyes were fixed in PBS-buffered 4% paraformaldehyde for 24 h and then in 30% sucrose/PBS. After dehydration, the eyecups were embedded in Optimal Cutting Temperature (OCT) compound (Tissue Tek; Sakura Finetek, Tokyo, Japan) for sectioning. Next, 10 µm serial sections were cut on a cryostat microtome and processed for immunodetection.

### TUNEL staining

Apoptotic cell death was analyzed with a TUNEL detection Kit (Roche, catalog number 11684795910) according to the manufacturer’s instructions. Cells were cultured in chamber slides and double labeled with DAPI (Molecular probes). Cells stained with both TUNEL and DAPI were considered positive for apoptosis.

## Results

**Figure 1:**
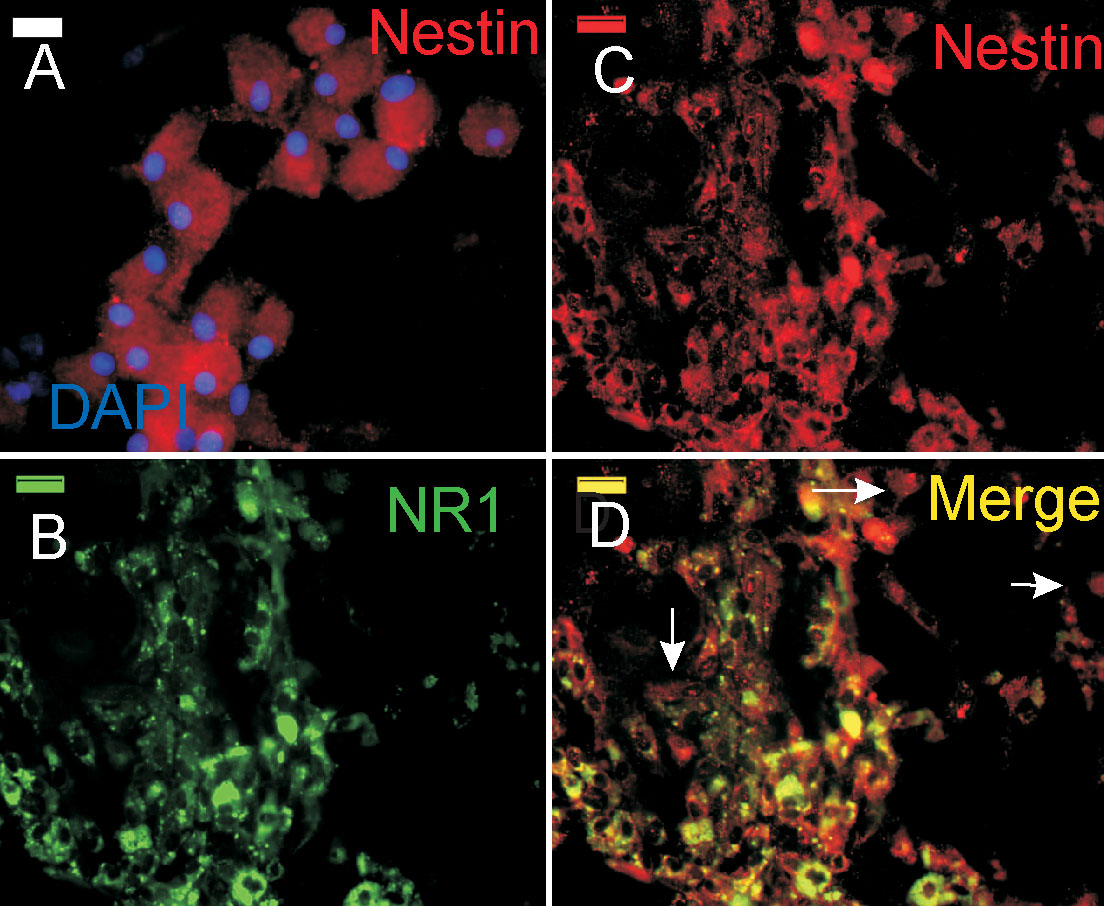
Postnatal Müller-derived progenitors express nestin and NR1. **A:** Photomicrograph shows cultured Müller-derived progenitors labeled with an antibody against nestin (red) and counterstained with DAPI (blue). **B-D:** Photomicrographs show cultured Müller-derived progenitors double-labeled with antibodies against NR1 (**B**), nestin (**C**), and overlay (**D**). Arrow indicates nestin immunopositive and NR1 inmmunonegative-labeled cells. Bar in each panel equals 50 μm.

Neurospheres were obtained from a primary culture of postnatal differentiated Müller cells after four days in proliferating conditions. The self-renewal capacity of the cells was assessed by dissociating them and replating them in the same culture conditions. Secondary and tertiary neurospheres could be obtained albeit with less frequency as the culture grew older (not shown). Characterization of isolated cells from these neurospheres by immunocytochemistry (Figure 1) revealed that more that 90% of these cells express nestin (Figure 1A). Next, we double-labeled for nestin and the NR1 subunit of the NMDA receptor (Figure 1B-D). Most of the nestin-positive cells colocalized with NR1 immunoreactivity. However, we also found that there were a few nestin-positive cells lacking NR1 expression (Figure 1D).

**Figure 2:**
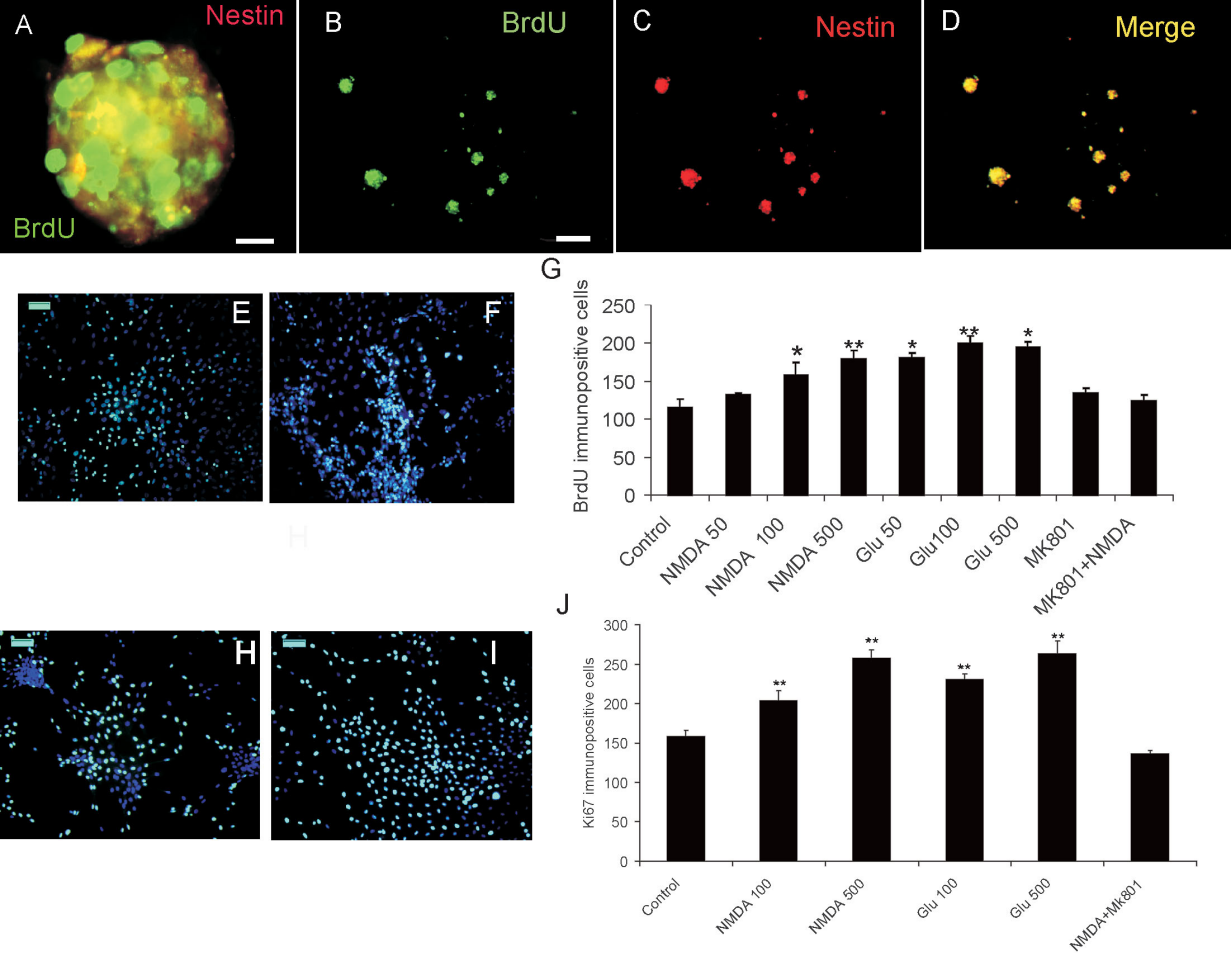
NMDA induces postnatal Müller-derived progenitor proliferation. **A-D:** Photomicrographs show neurospheres double-labeled with nestin and BrdU (A) or single-labeled with BrdU (**B**), nestin (**C**), and merge (**D**). **E-F**: Dissociated BrdU-labeled cells that received no treatment (**E**) or 500 μM NMDA (**F**). **G:** Quantification of BrdU positive cells after no treatment, 50–100–500 μM NMDA or glutamate, 50 μM antagonist MK801 or 100 μM NMDA plus antagonist. **H-I:** Photomicrographs show dissociated Ki67-labeled cells after no treatment (**H**) or 500 μM NMDA (**I**). **J:** Quantification of Ki67 positive cells after no treatment, 100–500 μM NMDA or glutamate, and 500 μM NMDA plus 50 μM MK801. Calibration bar equals 50 μm in **A**, **E**, and **H**, and 200 μm in **B**. Data represent mean±SEM of 300 cells counted on nonoverlapping fields in three experiments. Asterisk (*) indicates p<0.05 and double asterisk (**) p<0.01 compared to control.

To investigate if NMDA receptor-mediated mechanisms influence Müller cell-derived retinal progenitor proliferation, we double-labeled for BrdU and nestin. Figure 2 shows that, in neurospheres (Figure 2A-D) and in isolated cell preparations from these neurospheres (Figure 2E-F), nestin-positive cells proliferated, as indicated by BrdU incorporation.

The progenitor cells were treated with increasing concentrations of NMDA. Increased concentrations of glutamate were added in control cell wells; it has been previously described that glutamate increases cell proliferation in these cells [12]. The cells were then allowed to proliferate for five days and labeled 24 h before BrdU analysis. Quantification of these experiments in Figure 2G shows that, from 100 μM, NMDA is capable of inducing a statistically significant increase in cell proliferation although less effectively than glutamate. Blockade of NMDA receptors with 50 μM MK801 significantly decreased glutamate-induced proliferation. To exclude the possibility that BrdU could result from DNA repair in response to DNA damage we immunolabeled the proliferating cells with an anti Ki67 antibody and similar results were obtained (Figure 2H-J). These results support an active role of this subtype of glutamate receptors in the proliferation process. NMDA receptor activation induces transcription factor CREB phosphorylation in cultured Müller-derived progenitor cells.

**Figure 3:**
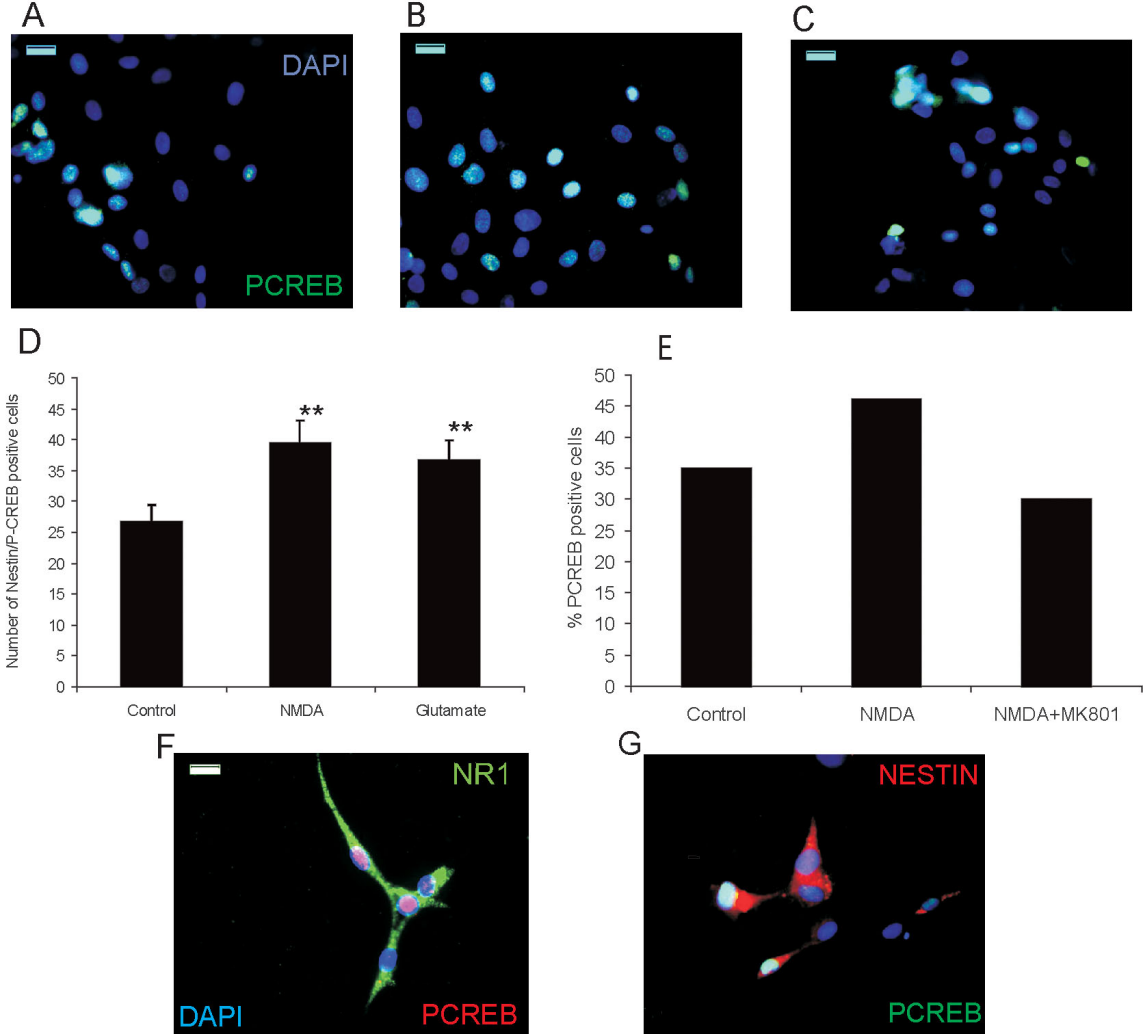
NMDA induces CREB phosphorylation in retinal progenitors. **A-C:** Photomicrographs show P-CREB labeled cells counterstained with DAPI after no treatment (**A**) and 100 μM NMDA (**B**), or glutamate (**C**). **D:** Graph shows the quantification of nestin-P-CREB double-positive cells in control, NMDA, or glutamate-treated cultures. Data represent mean±SEM of 100 cells counted on nonoverlapping fields in three experiments. Double asterisks (**) indicate p<0.05 compared to control. **E:** Graph shows the percentage of P-CREB positive cells after no treatment, 100 μM NMDA, or 100 μM NMDA plus 50 μM MK801. Data represent the percentage of P-CREB positive cells with respect to total number of cells counted on five nonoverlapping fields. **F-G:** Photomicrographs show NR1-P-CREB (**F**) and nestin-P-CREB double-labeled cells (**G**) counterstained with DAPI. Calibration bar equals 100 μm in **A-C** and 20 μm in **F**.

Next we wanted to take advantage of our purified Müller-derived progenitor cell culture to analyze the molecular signal cascade induced by NMDA receptor activation in these cells. We had previously described that NMDA receptor activation induces CREB phosphorylation at early times in differentiated Müller glia [13]. To test whether CREB phosphorylation is also induced in Müller-derived progenitor cells in culture, we examined the frequency of P-CREB immunoreactivity in cells that had been treated with 100 μM NMDA or 100 μM glutamate for 1 h. In these conditions, we found that there was a 47±7% increase in P-CREB immunoreactivity in NMDA- or glutamate-treated cells (Figure 3A-D). Complementary experiments shown in Figure 3E indicate that treatment of the cells with the NMDA receptor antagonist MK801 prevents NMDA-induced CREB phosphorylation.

Furthermore, colocalization of the NR1 subunit of the NMDA receptor and P-CREB was demonstrated by double immunolabeling with both antibodies (Figure 3F). Similarly, coimmunolabeling demonstrated colocalization of the progenitor marker nestin and P-CREB on the same cell, although with a different subcellular localization (Figure 3G).

**Figure 4:**
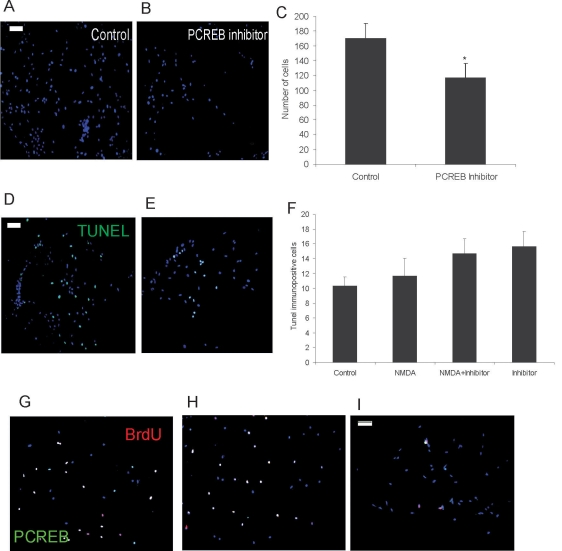
P-CREB blockade decreases NMDA-mediated cell proliferation. **A-C:** Photomicrographs show DAPI stained cells incubated in the absence (**A**) or presence (**B**) of 25 μM CREB inhibitor, for 48 h and cultured in fresh medium for additional 48 h. **C:** Graph shows quantification of control and CREB inhibitor treated cells. Data represent mean±SEM of cells counted on five nonoverlapping fields in three experiments. Asterisk (*) denotes p<0.05 compared to control. **D-F:** Photomicrographs show DAPI and TUNEL stained control (**D**) and CREB inhibitor (**E**) treated cells. **F:** Graph shows quantification of TUNEL positive cells in control, 100 μM NMDA, NMDA plus 25 μM CREB inhibitor or CREB inhibitor treated cells. Data are represented as in **C**. **G-I:** Photomicrographs show DAPI, BrdU, and P-CREB stained cells that were untreated (**G**) or treated with 100 μM NMDA (**H**) or NMDA plus CREB inhibitor (**I**). Calibration bar equals 50 μm in **A, D**, and **I**.

CREB is a pleiotropic transcription factor that has been involved both in cell proliferation and survival [15]. To further characterize the effect of CREB phosphorylation on cell proliferation, we cultured the cells in the presence of the CREB inhibitor 2-naphthol-AS-E-phosphate [15,21]. We analyzed for the presence of proliferating cells 48 h after treatment (Figure 4A-C). In these experiments, the cells were incubated in the presence or absence of inhibitor for 48 h, the inhibitor was washed away, and the cells were allowed to grow for an additional 48 h. Under these conditions, we found that P-CREB labeling was decreased by 90% (data not shown). Our results show that the number of proliferating cells decreases in CREB-inhibitor-treated cells as compared to control cultures (Figure 4A-C). To rule out the possibility that BrdU would be incorporated due to DNA repair processes and to determine whether the observed decrease in cell proliferation was due to an inhibitor-induced increase in cell death, we quantified TUNEL-immunopositive cells in cell cultures after the different treatments (Figure 4D-F). We found no statistical differences in the number of apoptotic cells among untreated or treated cultures. A further link between NMDA receptor activation, CREB phosphorylation, and Müller cell proliferation was obtained by cotreatment of the cells with NMDA and the CREB inhibitor for 24 h and coimmunolabeling for BrdU and P-CREB. These experiments indicated that partial inhibition of CREB phosphorylation led to a decrease in the number of of BrdU-immunopositive cells in NMDA plus CREB inhibitor-treated cultures (Figure 4G-I). Noteworthy, under these conditions, we also found several cells that were single labeled for either P-CREB or BrdU.

These results suggest that Müller-derived progenitor cells in culture respond to NMDA receptor activation, increasing the number of cells expressing phosphorylated CREB. In turn, this increase may contribute to the maintenance of a Müller-derived progenitor cell population.

**Figure 5:**
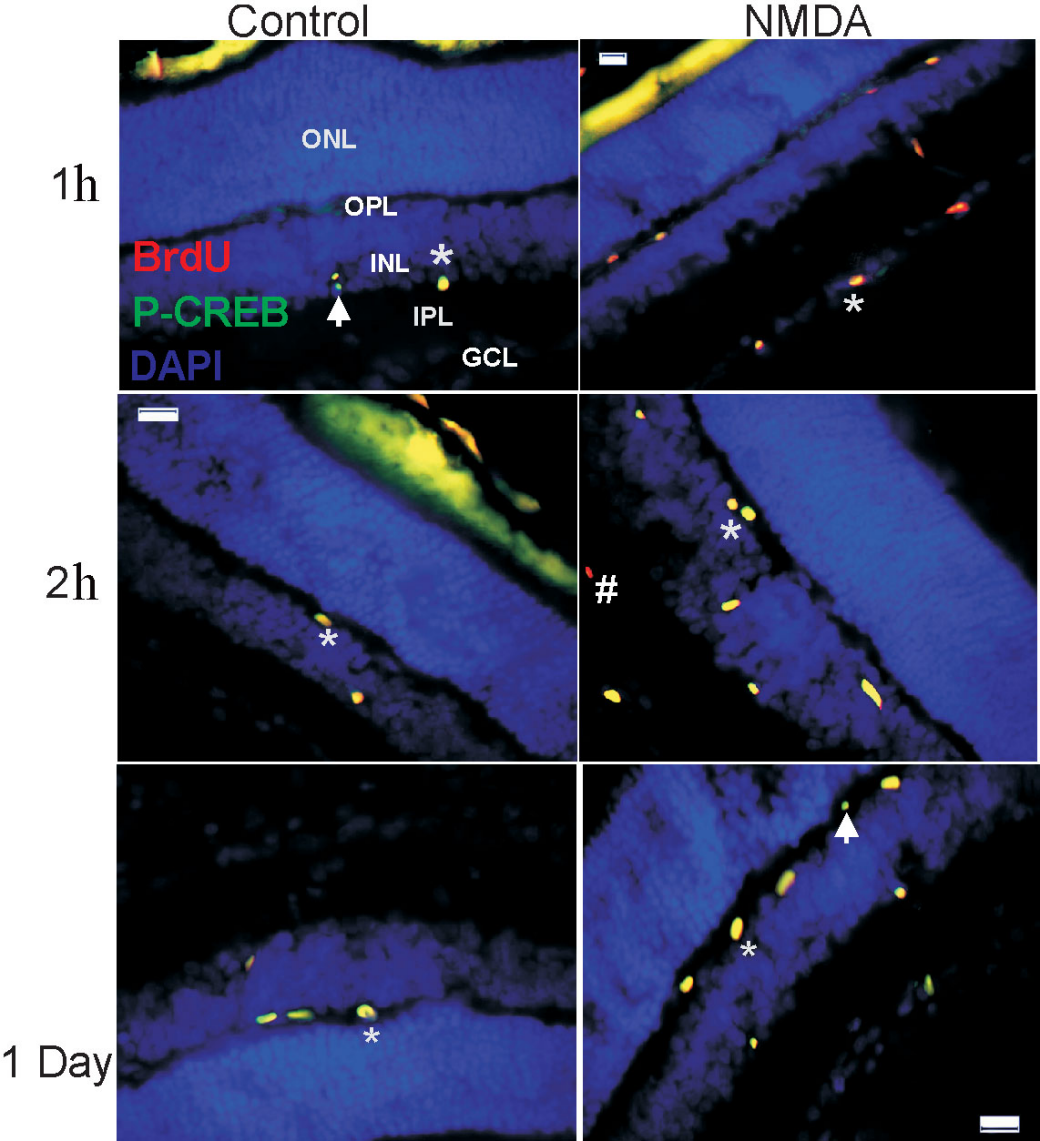
NMDA induces CREB phosphorylation in vivo. Photomicrographs show retinal sections collected from postnatal rats 1 h, 2 h, and 1 day after intravitreal injection of BrdU-saline (Control) or BrdU-NMDA (NMDA). The retinal sections were labeled with antibodies against P-CREB or BrdU and counterstained with DAPI. Abbreviations: ganglion cell layer (GCL); inner plexiform layer (IPL); inner nuclear layer (INL); outer plexiform layer (OPL); outer nuclear layer (ONL). Arrow indicates P-CREB immunopositive cells. Asterisk (*) denotes P-CREB-BrdU coimmunolabeled cells. Pound sign (#) marks BrdU immunopositive cells. Calibration bar denotes 50 μm in middle left panel.

Consequently we wanted to analyze the immunoreactivity pattern of phosphorylated CREB after NMDA injection in vivo at various times after treatment. We injected solutions containing 2 M NMDA/10 mM BrdU intravitreally and retinal sections were collected 1 h, 2 h, or one day after injection and immunolabelled for BrdU and P-CREB. Both P-CREB and BrdU were detectable at 1 h, 2 h, and one day after injection, and a small number of P-CREB positive cells were proliferating as indicated by BrdU labeling (Figure 5). The number of P-CREB positive cells was increased in NMDA-treated retinas. These results indicate that NMDA receptor activation induced CREB phosphorylation signal transduction pathway also in vivo in the retina, suggesting a role for this event in retinal proliferation.

## Discussion

In this study we present evidence that NMDA receptor activation induces postnatal Müller glia-derived retinal cell progenitor proliferation and transcription factor CREB phosphorylation. Numerous studies indicate that glutamate plays an important role in neural development and neurogenesis in the central nervous system in rodents [17,18,22] and humans [23]. There is now evidence that glutamate exerts contrasting effects on progenitor cell proliferation depending on the type of receptor used for signal transduction and the neurogenic area under study. Thus, the antiproliferative effect that glutamate has upon neuronal progenitors in the developing rodent cerebral cortex involves the AMPA/kainate subtype of ionotropic glutamate receptors [16,17], whereas NMDA receptors induce proliferation of neuronal progenitor cells from the rodent striatum [18].

Specifically in the retina, both functional glutamate transporters and receptors are expressed early during development [3]. Glutamate uptake by glutamate transporters is one of the most relevant and studied physiologic roles in differentiated Müller cells, having vast implications in retinal pathology studies. Compelling evidence demonstrates that a vast array of molecules and regulatory systems modulate glutamate clearance in these cells [24]. However, neither the expression profile of glutamate transporters nor the mechanisms of glutamate clearance have been explored in Müller glia-derived progenitor cells, and thus the contribution of these cells to glutamate uptake remains unclear and requires further analysis.

Noteworthy, the participation of glutamate receptors at early times during retinal development has been more profoundly analyzed. Research has revealed that activation of AMPA/kainate receptors induced rodent retinal progenitor cells to prematurely exit the cell cycle, leading to a dose-dependent decrease in cell proliferation while activation of NMDA receptors had no effect [4]. In contrast, it has been recently described that mature Müller glia in adult mice can be induced to dedifferentiate, migrate, and generate new retinal neurons by glutamate [12]. We demonstrate here that NMDA receptor activation contributes to proliferation of mature Müller glia-derived retinal cell progenitors in culture (Figure 2). The apparent discrepancy between previous reports could be attributed to the fact that, although the process of adult neurogenesis shares similar mechanisms with embryonic or postnatal neurogenesis [25], many aspects of the maturation process are different from what occurs during fetal development, specifically regarding the sequential expression of neurotransmitters receptors [5].

We have previously demonstrated that rat mature Müller glia responds to NMDA receptor activation, inducing changes in transcription factor expression and function [13,14]. Thus, we reported that NMDA receptor activation induces CREB phosphorylation in these cells [13]. We now demonstrate that, in rat postnatal Müller glia-derived retinal cell progenitors, CREB phosphorylation follows glutamate and NMDA treatment in culture (Figure 3 and Figure 4). However, two lines of evidence suggest that different populations of cells coexist in culture that are likely to be in a different maturation state and respond differently to a specific stimulus. We found that a small subset of Müller-derived retinal progenitors did not express the NR1 subunit of the NMDA receptor (Figure 1B-D), and we demonstrated that not all the cells respond to NMDA treatment by inducing CREB phosphorylation (Figure 3). Interestingly we observed that P-CREB immunopositive cells express also NR1 immunoreactivity (Figure 3F) and that blockade of NMDA receptor by MK801 treatment prevented NMDA receptor-induction of CREB phosphorylation (Figure 3E). These findings show that Müller-derived progenitors respond to NMDA receptor activation through the activation of CREB phosphorylation. Furthermore NMDA injections demonstrate that, also in vivo, CREB phosphorylation is induced after treatment (Figure 5).

Our results add to the previous reports on the involvement of CREB in the regulation of proliferation, differentiation, and survival of newborn neurons in different neurogenic areas of the rodent brain [15,26]. While CREB appears to induce adult progenitor proliferation and inhibit cell differentiation in the hippocampus [15], it is mostly involved in cell differentiation rather than proliferation in the subventricular zone of the olfactory bulb [26]. To our knowledge, we describe for the first time the expression of transcription factor CREB in proliferating postnatal Müller glia-derived retinal cell progenitors. The specific genes responsible for the action of CREB in postnatal Müller glia-derived retinal cell progenitors have not been defined. However, in a rat retinal neuron-glial progenitor cell line, CREB has been shown to mediate antiapoptotic effects induced by nitric oxide, supporting a role for CREB in progenitor survival in the retina [27].

A link between CREB phosphorylation and activation of members of the wingless-type MMTV integration site family (Wnt)/β−catenin signaling pathway that takes place during the developmental induction of myogenic genes has been demonstrated [28]. Interestingly, a member of the Wnt family, Wnt3a, has been shown to promote proliferation of Müller glia-derived retinal progenitors in the adult mammal damaged retina [29]. The identification of the molecular determinants of mature retinal progenitors such as transcription factor CREB and NMDA receptor-induced players should facilitate the control of growth and manipulation of progenitor cell cultures and the possible identification of the molecular mechanisms involved in progenitor self-renewal.
